# Supplementary material for: BCG-Vaccinated Children with Contact to Tuberculosis Patients Show Delayed Conversion of Mycobacterium tuberculosis-Specific IFN-γ Release
Source: Vaccines (Basel). 2023 Apr 17;11(4):855. doi: 10.3390/vaccines11040855 (PMC10146292; doi:10.3390/vaccines11040855)
Supplement: Supplementary file 1 [file vaccines-11-00855-s001.zip › vaccines-2290182-supplementary.pdf]

Supplementary Figure 1

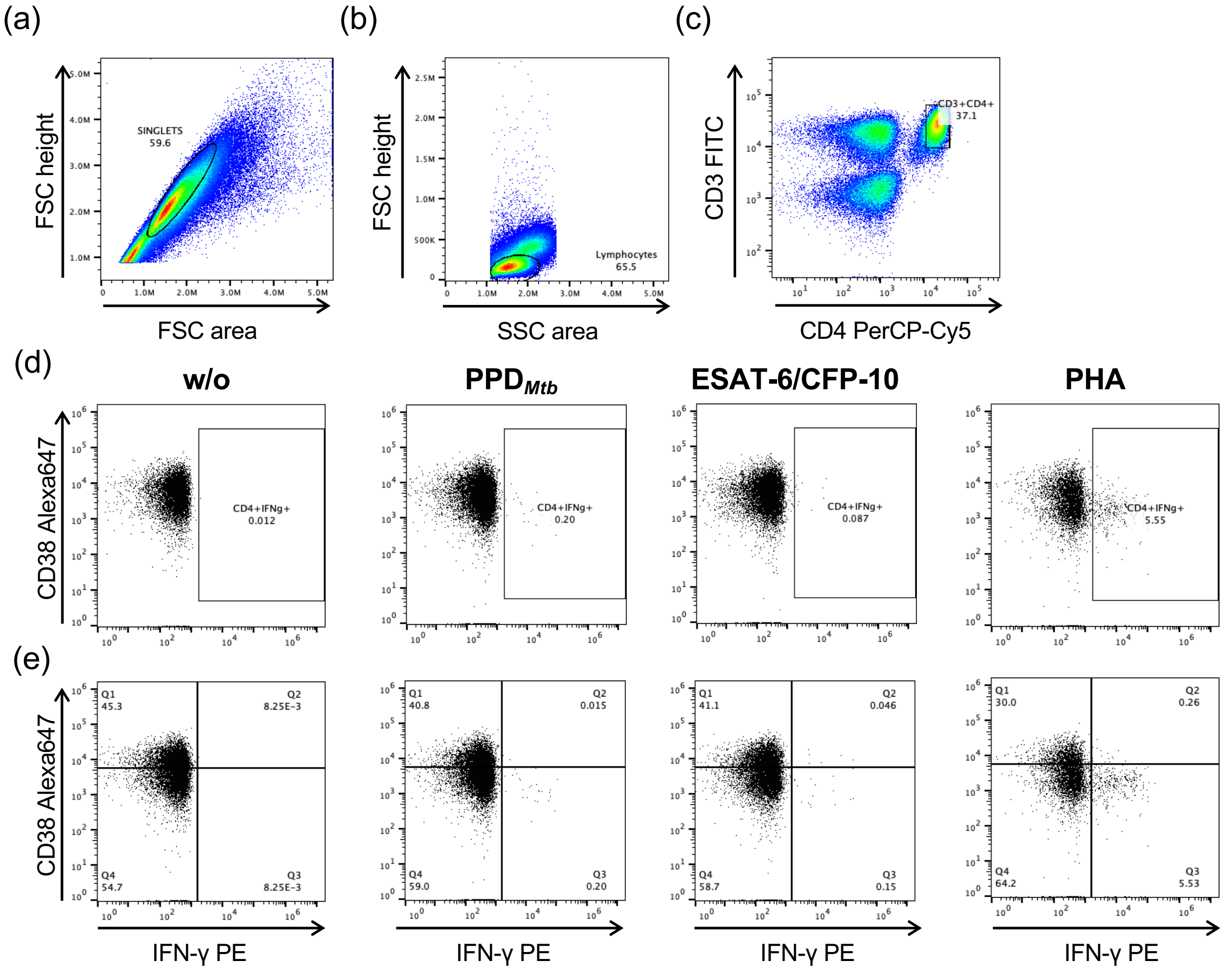

Flow cytometry gating strategy for quantification of IFN-γ^{+} CD4^{+} T-cell proportions and CD38 co-expression. The following steps for analysis are performed: (a) Exclusion of cell douplets by correlating forward scatter area and height measurements; (b) Gating of lympoid cells by size and granularity determination; (c) Gating of CD3^{+}/CD4^{+} T cells; (d) Quantification of CD3^{+}/CD4^{+} T cells expressing IFN-γ in differentially stimulated samples (mean of duplicates). Unstimulated samples (w/o) were subtracted from PPD$_{Mtb}$, ESAT-6/CFP-10, and phytohemagglutinin (PHA) stimulated samples; (e) Proportions of CD38^{+} CD3^{+}/CD4^{+} T cells were calculated by dividing the number of cells from the upper right and the lower quadrants (background numbers are subtracted as for (d)) and multipled with 100 to get proportions.
